# Regulatory mechanisms of nutritional factors on melanin deposition in Taihe Silky Fowls: review

**DOI:** 10.5713/ab.250725

**Published:** 2026-03-11

**Authors:** Langlang Fu, Wenbin Dai, Shilei Zhou, Chengwei Wang

**Affiliations:** 1 College of Life Science, Jiangxi Science and Technology Normal University, Nanchang, China

**Keywords:** Melanin, Nutritional Factor, Regulatory Mechanism, Taihe Silky Fowl

## Abstract

The black pigmentation trait in Taihe Silky Fowls results from melanin deposition produced by widely distributed melanocytes within their bodies. The degree of melanin deposition, termed melanization, not only determines their external characteristics but also critically influences their nutritional properties and medicinal value. Nutritionally, Taihe Silky Fowls with high melanization are rich in trace elements such as iron and zinc, along with various vitamins, conferring high nutritional value. Medicinally, according to traditional Chinese medicine theory, highly melanized Taihe Silky Fowls possess therapeutic effects including nourishing the liver and kidney, replenishing Qi, and enriching the blood. Specifically, they improve liver and kidney health, promote blood production and circulation, and enhance immune capacity. This paper comprehensively reviews the molecular mechanisms of melanin biosynthesis and deposition. It specifically analyzes how particular nutrients, including amino acids, vitamins, and minerals, regulate melanin deposition in Taihe Silky Fowls at the level of cellular pathways, while also discussing their appropriate dietary supplementation levels in production practices. The aim of this review is to deepen the understanding of the mechanisms underlying melanin trait formation in Taihe Silky Fowls and to provide theoretical support for the scientific optimization of their dietary nutritional formulations.

## INTRODUCTION

The Taihe Silky Fowl (*Gallus gallus domesticus*), originating from the Wushan foothills in Taihe County, Jiangxi Province, South China, is renowned for the ten distinctive characteristics: black bones, black skin, black flesh, crest-shaped head, five-toed feet, a mulberry comb, greenish earlobes, feathered shanks, silky plumage, and beard-like feathers [1,2]. They are also celebrated for their high nutritional and medicinal value. As a premium local breed with multi-purpose applications, Taihe Silky Fowls serve medicinal, culinary, egg production, and ornamental functions. Compared to ordinary broiler chickens, Taihe Silky Fowls exhibit significantly higher levels of melanin in their various tissues. The nourishing efficacy of these fowls as a tonic positively correlates with their melanin content, meaning that higher melanin levels correspond to superior medicinal effects [3]. The abundant melanin found in Taihe Silky Fowl tissues primarily exists as a biological polymer in the form of melanin-protein complexes. This melanin exerts multi-faceted health-promoting effects through dietary intake in humans. Melanin in Taihe Silky Fowl exerts potent antioxidant activity as its primary biological function, which is enabled by its unique polymeric indolequinone structure that efficiently scavenges excessive reactive oxygen species (ROS) within the body, such as superoxide anion (O_2_•^−^) and hydroxyl radicals (•OH) [4,5]. This effectively protects cellular membranes, proteins, and DNA from oxidative damage, significantly slowing cellular aging and the decline of tissue function [6,7]. Concurrently, melanin in Taihe Silky Fowl exerts its unique bidirectional immunomodulatory and anti-inflammatory effects: it enhances innate immune defenses by boosting macrophage phagocytic capacity and natural killer (NK) cell activity, while also mitigating chronic low-grade inflammation and maintaining immune system homeostasis through suppressing the overactivation of key inflammatory signaling pathways such as NF-κB, thereby reducing the production of pro-inflammatory cytokines including tumor necrosis factor alpha (TNF-α) and interleukin 6 (IL-6) [8–10]. Additionally, this breed-specific melanin demonstrates unique potential for supporting metabolic balance; its antioxidant properties contribute to improved insulin sensitivity, exerting a beneficial influence on the maintenance of glucose homeostasis [11].

The health benefits of melanin in Taihe Silky Fowls derive from its unique physicochemical properties, specifically free radical scavenging [12] and metal chelation [13], combined with its complex formation with proteins. This enables melanin not only to eliminate harmful substances but also to deliver comprehensive health benefits at molecular and cellular levels through modulation of key signaling pathways including Nrf2 and NF-κB. Melanin synthesis occurs within melanocytes followed by extracellular transport and tissue deposition. In vertebrates, melanocytes originate from neural crest cells that differentiate into melanoblasts during early embryogenesis. These melanoblasts migrate through the somitic mesoderm to the ectoderm, establishing widespread distribution of epidermal melanocytes. The intensity of black pigmentation in Taihe Silky Fowls primarily depends on melanin type and concentration [14,15]. Melanin deposition is genetically regulated. Recent studies in other species have revealed that melanogenesis is precisely modulated at the post-transcriptional level through mechanisms involving ceRNA networks as well as specific non-coding RNAs, such as miRNAs and circRNAs [16–18]. However, melanin deposition is strictly genetically regulated while remaining environmentally sensitive. This review systematically examines melanin biosynthesis in Taihe Silky Fowls and nutritional modulation of melanin deposition, providing theoretical foundations for future research.

## MOLECULAR MECHANISMS OF MELANIN SYNTHESIS

### The melanin biosynthetic pathway

Melanins, phenolic biopolymers composed of tyrosine and related phenolic compounds [19], are widely distributed in the hair, skin, muscle tissues, and various internal organs of livestock and poultry [20]. Generally, melanins are classified into four types: eumelanin, pheomelanin, allomelanin, and neuromelanin. Among these, the melanin in Taihe Silky Fowls primarily consists of two chemical forms [21]: Eumelanin, formed by the oxidation of tyrosine-derived indole monomers, specifically 5,6-dihydroxyindole (DHI) and 5,6-dihydroxyindole-2-carboxylic acid (DHICA), lacks sulfur atoms and exhibits brown to black coloration. Pheomelanin, generated through the oxidation of cysteinyldopas adducts to form benzothiazine derivatives, contains sulfur atoms and displays yellow to reddish-brown hues. The intensity of black pigmentation in Taihe Silky Fowls is predominantly determined by the relative deposition ratios of eumelanin and pheomelanin. The melanin content of Taihe Silky Fowls is significantly higher than that of Yanjin silky fowls, Muchuan silky fowls and Jiuyuan black chickens, and slightly higher than that of Chishui silky fowls. The possible reason for its significant advantage in melanin content is the variation of the TYR gene and its differential expression characteristics in different tissues [22]. Zu et al [23] indicated that the difference in blackness between Chishui silky fowls and Taihe Silky Fowls might be determined by the C921T variation in exon 1 of the TYR gene.

The biosynthesis of melanin in Taihe Silky Fowls begins with the hydroxylation of tyrosine (Tyr) catalyzed by tyrosinase (TYR) to form L-3,4-dihydroxyphenylalanine (L-DOPA), which is subsequently oxidatively dehydrogenated by TYR to generate dopaquinone (DQ). This step serves as the rate-limiting and critical regulatory node in melanogenesis [24]. Depending on the presence or absence of thiol compounds (e.g., cysteine, Cys, or glutathione, GSH) in the microenvironment, DQ diverges into two distinct pathways: In the presence of Cys/GSH, DQ conjugates with thiols to form cysteinyl-DOPA (Cys-DOPA) or glutathione-DOPA (GSH-DOPA), which undergo cyclization to produce benzothiazine intermediates that are oxidized and polymerized into pheomelanin. Conversely, under thiol-deficient conditions, DQ spontaneously cyclizes to leucodopachrome (DC), which is rapidly oxidized to dopachrome [25]. Dopachrome is then isomerized via TYR-related protein-2 (TYRP2) to yield DHICA. The DHICA undergoes decarboxylation and oxidation mediated by TYR-related protein-1 (TYRP1), ultimately polymerizing into eumelanin. Additionally, dopachrome may spontaneously decarboxylate to form DHI, which is directly oxidized and polymerized by TYR into eumelanin. The synthesis ratio of pheomelanin to eumelanin is predominantly regulated by thiol availability, with high thiol concentrations promoting pheomelanin production and low thiol conditions favoring eumelanin synthesis. TYR activity governs the total melanin output, while the coordinated enzymatic functions of TYRP1 and TYRP2 are essential for eumelanin biosynthesis [26].

### Signaling pathways regulating melanin synthesis

**Figure 1:**
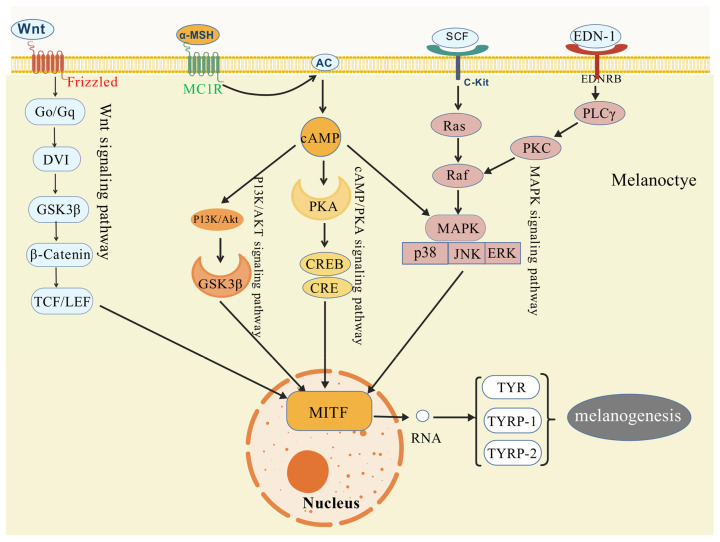
Diagram of melanogenesis signaling. Melanin production is regulated by various intracellular signalling pathways, such as the cAMP/PKA signalling pathway, the MAPK signalling pathway, and the Wnt signalling pathway, through which signals are transmitted to melanocyte inducing transcription factor (MITF), thereby regulating the gene expression of tyrosinase (TYR), tyrosinase-related protein-1 (TYRP1) and tyrosinase-related protein-2 (TYRP2), and affecting melanin formation. Data from Feixuan et al [110]. Created with BioGDP.com [111]. cAMP, cyclic AMP; PKA, protein kinase A.

Melanogenesis in Taihe Silky Fowls proceeds through four stages: melanosome formation, melanosome maturation, melanin synthesis, and melanin transport, each precisely regulated by specific signaling molecules and components [27]. Beyond such intracellular regulation, melanin production may also be influenced by intercellular communication, as evidenced in rabbits where dermal papilla cell-derived exosomes inhibit melanogenesis [28], suggesting a potential parallel in Taihe Silky Fowls. The production of melanin in Taihe Silky Fowls is governed by multiple intracellular signaling pathways [29], including the cyclic AMP (cAMP)/protein kinase A (PKA) pathway [30], MAPK pathway [31], and Wnt pathway [32]. These pathways converge on the microphthalmia-associated transcription factor (MITF), which regulates the expression of *TYR*, *TYRP1*, and *TYRP2* genes [33,34]. The mechanisms are detailed below (Figure 1).

#### Wnt signaling pathway

Wnt, a family of secreted glycoproteins, binds to Frizzled receptors to activate downstream signaling. This pathway inhibits glycogen synthase kinase 3 beta (GSK-3β) activity, leading to cytoplasmic accumulation and nuclear translocation of β-catenin. Nuclear β-catenin forms a complex with T-cell factor (TCF) and lymphoid enhancer-binding factor (LEF), directly binding to the MITF promoter to enhance its transcriptional activity [35].

#### PI3K/AKT signaling pathway

Upon binding of α-melanocyte-stimulating hormone (α-MSH) to the melanocortin-1 receptor (MC1R) on the melanocyte membrane, adenylate cyclase (AC) is activated to generate cAMP. cAMP subsequently initiates the activation of PI3K, which in turn promotes the phosphorylation and activation of AKT. Activated AKT inhibits glycogen synthase kinase 3β (GSK3β), thereby relieving GSK3β-mediated degradation control over MITF. Consequently, the nuclear level of MITF is stabilized and elevated. As a key transcription factor in melanin synthesis, MITF further drives the transcriptional expression of genes encoding critical melanogenic enzymes such as TYR, TYRP-1, and TYRP-2, ultimately promoting melanogenesis [36]. The PI3K/AKT signaling pathway also cooperates with other pathways, including the cAMP/PKA pathway, to collectively regulate melanin synthesis in melanocytes.

#### Cyclic AMP/protein kinase A signaling pathway

The MC1R, a central regulator of epidermal melanogenesis, serves as the receptor for α-MSH. Upon α-MSH binding, MC1R activates G protein-coupled AC, elevating intracellular cAMP levels. cAMP activates PKA, which phosphorylates cAMP-response element binding protein (CREB). Phosphorylated CREB binds to cAMP-response elements (CREs) in the MITF promoter, upregulating MITF expression. Additionally, PKA phosphorylates GSK-3β to stabilize MITF, amplifying its transcriptional activity [37].

#### MAPK signaling pathway

Stem cell factor (SCF) binding to the C-Kit receptor tyrosine kinase activates the Ras-Raf-MEK-ERK cascade. Activated ERK phosphorylates MITF at Ser73, promoting its ubiquitination and degradation, thereby suppressing melanogenesis. Conversely, ERK synergizes with the cAMP pathway to enhance CREB activity, indirectly upregulating *MITF*. Furthermore, p38 MAPK stabilizes MITF and enhances its transcriptional activity, positively regulating melanin synthesis [38]. Additionally, endothelin-1 (EDN-1) binds to the endothelin B receptor (EDNBR), activating phospholipase Cγ (PLCγ) to generate diacylglycerol (DAG) and activate protein kinase C (PKC). Activated PKC phosphorylates Raf kinase, triggering the MAPK cascade to modulate MITF activity and melanogenesis.

## REGULATORY MECHANISMS OF MELANIN DEPOSITION BY NUTRITIONAL FACTORS

### Amino acids

#### Tyrosine

**Figure 2:**
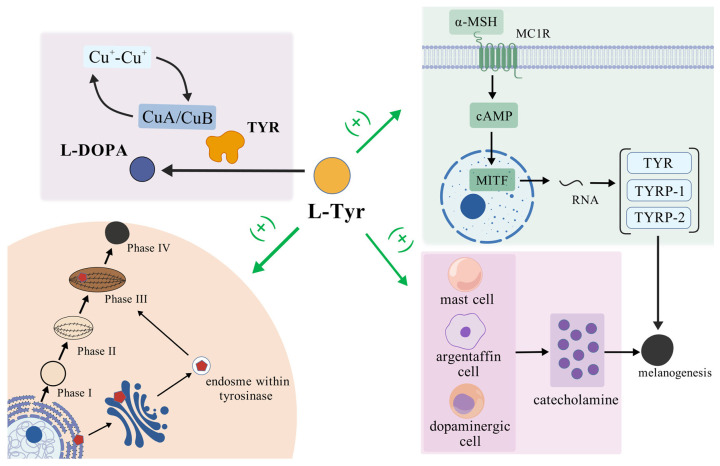
Diagram of the tyrosine regulatory mechanism. L-Tyrosine (L-Tyr) specifically binds to the active centre (CuA/CuB) of the binuclear copper cluster of tyrosinase, inducing conformational rearrangement of the enzyme, and its metabolites also promote Cu^+^-Cu^+^ re-oxidation, thereby increasing enzyme activity; at the same time, L-Tyr regulates the proliferative activity induced by α-melanocyte-stimulating hormone (α-MSH), and regulates melanin formation by influencing gene expression; furthermore, L-Tyr also promotes the melanin synthesis and assembly of melanosomes and serves as a raw material for the synthesis of catecholamines in pro-silver cells and other cells, promoting melanin synthesis. Data from Xiao [42]. Created with BioGDP.com [111].

L-Tyrosine acts as a positive regulator of melanogenesis, with its regulatory effects varying depending on species, cell genotype, and environmental conditions. The influence of L-tyrosine on melanin synthesis differs across cell types through the following mechanisms: (1) Direct participation in the synthesis of both melanin and catecholamines is achieved by serving as their precursor through substrate provision [39,40]; (2) Activation of TYR in a dose- and time-dependent manner, thereby modulating melanin synthesis; (3) Promotion of premelanosome formation; (4) Indirect regulation of pigmentation by altering cell proliferation activity and melanogenic intensity induced by α-MSH (Figure 2).

L-Tyrosine serves not only as the direct substrate for melanin synthesis but also as a precursor for catecholamine production in argentaffin cells, dopaminergic cells, and mast cells. These cells synthesize and secrete catecholamines, such as dopamine, epinephrine, and norepinephrine, by uptake of L-tyrosine. These catecholamines further act as precursors to promote melanin synthesis [41,42]. Specifically, dopamine binds to the β2-adrenergic receptor (β2-AR) on the surface of melanocytes, activating the G protein-coupled signaling pathway: β2-AR interacts with heterotrimeric G proteins (composed of α, β, and γ subunits), inducing GTP binding and dissociation of the Gα subunit. This subsequently activates AC, which catalyzes the conversion of ATP to cAMP. As a second messenger, cAMP activates PKA, whose catalytic subunit phosphorylates specific sites on MITF, enhancing its transcriptional activity. This ultimately upregulates the expression of key melanogenesis-related genes, including TYR, TYRP1, and TYRP2. Tyrosinase catalyzes the oxidation of L-tyrosine to DOPA, followed by stepwise polymerization through intermediate products such as DQ and indolequinone to form melanin. Si et al [43] demonstrated a positive correlation between tyrosine concentration and mast cell numbers. Supplementing the daily diet of Taihe Silky Fowls with 0.8% additional tyrosine resulted in the peak thymic mast cell count.

L-Tyrosine, the natural substrate of TYR, enhances enzymatic activity through multiple mechanisms [44]. Its key mechanism involves specific binding to the binuclear copper cluster active site (CuA/CuB), inducing conformational rearrangement to optimize the catalytic pocket topology. During binding, the phenolic hydroxyl oxygen forms coordination bonds with the copper ions, stabilizing the histidine ligand environment and facilitating electron transfer, thereby preserving the essential μ-η^2^:η^2^ peroxo-bridged structure required for TYR activity. Although L-tyrosine initiates the hydroxylation catalytic cycle, its primary metabolite, L-DOPA, significantly boosts the enzyme turnover rate by efficiently reoxidizing the reduced enzyme form (Cu^+^-Cu^+^) generated indicates that beyond its substrate role, L-tyrosine enhances catalytic efficiency synergistically through conformational stabilization, microenvironment optimization, and L-DOPA mediated enzyme regeneration. Zhao et al [45–47] demonstrated that dietary additional tyrosine supplementation increased melanin content in Taihe Silky Fowl tissues up to a tissue- and age-specific threshold, beyond which efficacy diminished. Specifically, for Taihe Silky Fowls at different age stages, there are significant differences in the dietary tyrosine levels corresponding to the peak melanin content in tissues such as the heart, liver, muscle, and skin; moreover, the tyrosine levels corresponding to the peak value of the same tissue vary with different age stages. These results confirm the existence of a threshold for the melanin promoting effect of tyrosine, beyond which efficacy diminishes. Therefore, achieving optimal melanin deposition in specific tissues requires precise dietary tyrosine level adjustments based on the age of the Taihe Silky Fowls.

L-Tyrosine induces premelanosome formation, promoting melanosome synthesis and assembly [48]. Melanogenesis in Taihe Silky Fowls involves four sequential stages: melanosome formation, melanosome maturation, melanin synthesis, and melanin transport [49]. Melanosome maturation progresses through four consecutive morphological phases, characterized by distinct ultrastructural features and melanin content. Stages I and II represent premelanosomes, which display spherical or ovoid morphology, lack melanin deposition, and exhibit minimal enzymatic activity. Stage I melanosomes contain intraluminal vesicles, while Stage II premelanosomes develop a fibrillar protein matrix. During Stage III, melanosomes adopt an elliptical shape, and TYR and TYRP1 are transported into their lumen via Rab32/38 GTPases, initiating melanin deposition. Stage IV melanosomes appear as fully opaque granules, where complete pigment deposition obscures the underlying structure, and melanosomes lose nearly all melanogenic activity [50]. Melanin transport primarily refers to the process by which melanosomes, the carriers of melanin, are transferred from melanocytes to surrounding keratinocytes [29]. Upon entering the target cells, the melanosomes undergo enzymatic degradation, releasing melanin. Gao et al [51]. demonstrated that adding 0%, 0.2%, 0.4%, 0.6%, or 0.8% additional tyrosine to the diet of Guyuan silky fowls, compared with the control group, promoted an increase in melanosome number in all experimental groups, leading to elevated melanin release.

L-Tyrosine levels can modulate both α-MSH-induced proliferative activity and the extent of melanogenesis in melanocytes, consequently increasing melanin content [52,53]. In Taihe Silky Fowls, the MC1R forms a complex with α-MSH, activating the cAMP signaling pathway via G proteins. This cascade subsequently activates the CREB, ultimately upregulating the expression of the MITF gene. Activated MITF promotes the expression of TYR, TYRP1, and TYRP2, thereby regulating melanin synthesis rates [51]. Additionally, α-MSH enhances melanocyte proliferation and differentiation, creating a more favorable cellular microenvironment for melanogenesis [54]. Xiong et al [55] indicated that compared with the control group, α-MSH at concentrations of 2.5, 5, and 10 mg/L promoted the proliferation of *in vitro* cultured Taihe Silky Fowl skin melanocytes. It also increased *MC1R* gene expression, cAMP content, and TYR activity, consequently enhancing melanin synthesis. Notably, compared with the 5 mg/L and 10 mg/L groups, 2.5 mg/L α-MSH elicited a more pronounced stimulatory effect on all these parameters.

#### Phenylalanine

Phenylalanine, an essential amino acid with an aromatic benzene ring side chain, serves as the direct precursor for L-tyrosine in Taihe Silky Fowls. This conversion is catalyzed by phenylalanine hydroxylase (PAH), a monooxygenase utilizing tetrahydrobiopterin (BH_4_) as a cofactor. The resulting L-tyrosine is the primary precursor for melanin biosynthesis. Systemic L-tyrosine in Taihe Silky Fowls originates from two main sources: (1) Active absorption of dietary L-tyrosine via the gastrointestinal tract; (2) PAH catalyzed hydroxylation of dietary or endogenous phenylalanine in the liver. The liver, due to its high PAH enzymatic activity, is the principal organ supplying L-tyrosine systemically and plays a central role in maintaining the peripheral tyrosine pool [56,57]. Thus, the effective phenylalanine concentration directly influences the bioavailability of L-tyrosine available for melanin synthesis, thereby impacting melanin production. Li et al [58] indicates that the melanin-stimulating effect of dietary additional phenylalanine supplementation in Taihe Silky Fowls exhibits a threshold. Peak melanin levels occurred at specific concentrations of dietary additional phenylalanine supplementation: for 5- to 8-week-old fowls (6.79 to 11.49 g/kg phenylalanine), breast muscle, leg muscle, and skin melanin peaked at 9.14 g/kg; for 9- to 12-week-old fowls (6.37 to 10.19 g/kg phenylalanine), skin melanin peaked at 8.28 g/kg. The researchers hypothesized that when dietary additional phenylalanine levels exceed the requirement, phenylalanine may be primarily diverted to the deamination pathway rather than the tyrosine synthesis pathway, and the excessive intermediate metabolites produced by the deamination pathway may consequently inhibit melanin synthesis in breast muscle.

Beyond providing an essential substrate, phenylalanine indirectly influences pigmentation by regulating key enzyme expression in the melanin synthesis pathway. PAH, the rate-limiting enzyme converting phenylalanine to tyrosine, directly determines tyrosine yield through its gene expression level, thereby controlling melanin synthesis efficiency [59]. Fluctuations in phenylalanine concentration activate amino acid sensing mechanisms (e.g., the mTORC1 pathway) on the lysosomal surface, promoting mTORC1 complex recruitment and activation. Activated mTORC1 signaling can indirectly modulate PAH expression or activity via transcriptional, translational, or post-translational regulation, thus precisely controlling the conversion efficiency of phenylalanine to tyrosine. This amplifies its control over tyrosine supply and melanogenesis. Critically, phenylalanine/tyrosine metabolic flux modulates the expression or activity of MITF, the melanocyte-specific transcription factor. Li et al [60]. demonstrated that Taihe Silky Fowls require 18.49% to 26.5% higher dietary phenylalanine+tyrosine for optimal growth performance compared to white-shell laying hens with similar growth rates.

#### Tryptophan

Although tryptophan has less direct regulatory effects on melanin synthesis in Taihe Silky Fowls compared to tyrosine and phenylalanine, its dietary levels may indirectly influence melanogenesis through biochemical pathways including amino acid metabolic competition. The supply of dietary Tyr and Phe must be enough to maximize the melanin biosynthesis in the body of Tai he silky fowl, whereas excessive supply of Trp can influence the absorption and metabolism of Tyr and Phe and thus decrease the melanin synthesis [61]. The inhibitory effect of excessive dietary tryptophan on Tyr and Phe metabolism stems from systemic resource competition. During absorption and transport, tryptophan competes with Tyr and Phe for the LAT1 transporter via shared aromatic properties, directly reducing their intracellular availability. Within metabolic pathways, the aromatic ring structure of tryptophan competitively occupies the active site of PAH, increasing the Km value for the Phe→Tyr conversion. Concurrently, it interferes with the substrate-binding domain of TYR, impeding the Tyr→DQ oxidation process. Tryptophan oxidation products further deplete molecular oxygen and copper ion cofactors essential for TYR activity. Additionally, at the cofactor level, the kynurenine pathway, which accounts for 95% of tryptophan metabolic flux, continuously consumes NADPH to sustain indoleamine 2,3-dioxygenase (IDO) activity. This conflicts with PAH catalysis, which equally depends on NADPH and BH_4_ for hydroxylation. Compounded by tryptophan hydroxylase (TPH) competing for BH_4_ during 5-hydroxytryptamine (5-HT) synthesis, these processes ultimately cause a sharp decline in PAH efficacy due to cofactor deficiency. Li et al [61] revealed through dietary supplementation ratio experiments that the melanin content in the pectoral muscle of Taihe Silky Fowls peaks when the dietary supplementation ratio of Tyr, Phe, and Trp is 87:121:14. Therefore, precise control of the dietary supplementation ratio among tryptophan, tyrosine, and phenylalanine is critical to balance amino acid metabolic competition and optimize melanin synthesis in Taihe Silky Fowls.

**Figure 3:**
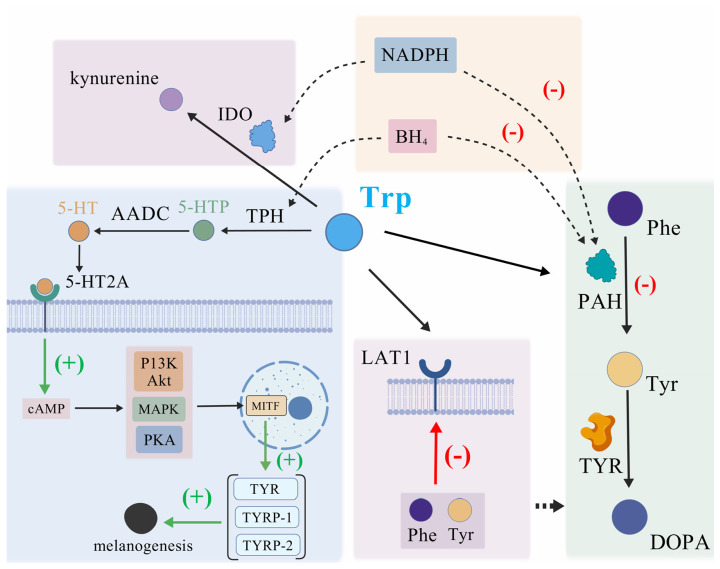
Diagram of the tryptophan regulatory mechanism. Tryptophan (Trp) undergoes a series of transformations catalysed by enzymes such as tryptophan hydroxylase (TPH), triggering the cAMP-PKA signalling cascade to promote melanin synthesis. Concurrently, Trp competes with Tyr and Phe for entry into the intracellular L-type amino acid transporter 1 (LAT1) transporter, influencing amino acid metabolism and competitive biochemical pathways thereby affecting melanin formation. Furthermore, Trp competes for the active site of phenylalanine hydroxylase (PAH) and influences melanin synthesis by affecting cofactors such as NADPH and BH_4_. Data from Agus et al [112]. Created with BioGDP.com [111]. cAMP, cyclic AMP; PKA, protein kinase A.

Tryptophan metabolism is essential for maintaining systemic homeostasis in Taihe Silky Fowls, providing a favorable microenvironment for melanin synthesis. Tryptophan is primarily metabolized through three pathways [62]: (1) Kynurenine Pathway (KP): Approximately 95% of tryptophan is catalyzed by indoleamine IDO to produce kynurenine. Its derivatives, such as quinolinic acid, participate in immune regulation and oxidative stress responses, potentially influencing melanocyte microenvironments and pigmentation indirectly; (2) Serotonin (5-HT) Pathway: Tryptophan is hydroxylated by TPH to form 5-hydroxytryptophan (5-HTP), which is rapidly decarboxylated by aromatic L-amino acid decarboxylase (AADC) to generate 5-HT. By activating 5-HT2A receptors on melanocytes, 5-HT initiates cAMP-PKA signaling cascades, promoting melanocyte proliferation, differentiation, and TYR activity, thereby enhancing melanin synthesis [63] (Figure 3); (3) Tryptamine-Indole Pathway: A small fraction of tryptophan is decarboxylated to produce tryptamine, which is further converted to melatonin or indole derivatives. These metabolites regulate circadian rhythms and antioxidant defenses, potentially preserving melanocyte function by alleviating oxidative damage [64,65]. Xiao [42] found that argentaffin cells, dopaminergic cells, and mast cells in Taihe Silky Fowls synthesize and secrete 5-HT. An increase in the number of these three cell types elevates 5-HT production. Specifically, mast cells are important immune cells in Taihe Silky Fowls that secrete various chemical mediators including catecholamines and 5-HT [41], and both catecholamines and 5-HT can directly act as substrates for melanin synthesis and participate in melanin production upon catalysis by TYR [66].

### Vitamins

#### Vitamin C

Vitamin C, also known as ascorbic acid, exhibits physiological functions such as photoprotection, melanin reduction [67], free radical scavenging, and immune regulation in living organisms. Vitamin C regulates melanin synthesis primarily through two key biochemical pathways: direct inhibition of TYR activity and interference with melanin oxidation-polymerization. Regarding TYR inhibition, Vitamin C acts as a potent reducing agent, competitively chelating the copper ions within the enzyme’s active site. Tyrosinase, the rate-limiting enzyme in melanogenesis, possesses an active site containing two copper ions (Cu^2+^), each coordinated with histidine residues forming an oxygen-carrier motif. The enediol structure of Vitamin C binds these copper ions, altering the enzyme’s conformation and thereby reducing its catalytic efficiency in converting tyrosine to DQ [68,69]. Concerning interference with melanin oxidation-polymerization, Vitamin C donates electrons to neutralize radical intermediates. This reduces DQ back to leucodopa, a non-pigmentary precursor, blocking its conversion to dopachrome and thus suppressing the melanin chain reaction at its origin [70].

**Figure 4:**
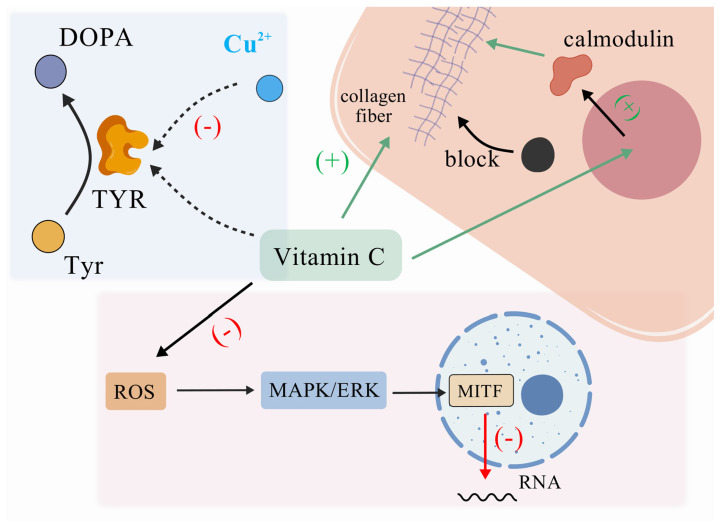
Diagram of the Vitamin C regulatory mechanism. Vitamin C competitively binds to the copper ion site within the active centre of tyrosinase, thereby affecting enzyme activity. Concurrently, adequate Vitamin C promotes the ordered arrangement of collagen and upregulates calmodulin expression, enhancing melanocyte adhesion and consequently reducing the transfer efficiency of melanosomes. Furthermore, Vitamin C neutralises reactive oxygen species (ROS) and inhibits the activation of the MAPK/ERK signalling pathway, thereby diminishing tyrosinase synthesis. Data from Sanadi and Deshmukh [72]. Created with BioGDP.com [111].

Vitamin C indirectly influences melanin deposition by regulating collagen metabolism, melanin transfer mechanisms, and scavenging ROS. In skin tissue, adequate Vitamin C promotes the organized arrangement of collagen fibers [71], forming a compact dermal-epidermal junction structure. This structure physically impedes the migration of melanosomes to keratinocytes. Concurrently, Vitamin C enhances adherens junctions between melanocytes and keratinocytes by upregulating cadherin expression, reducing dendritic projection extension, thereby decreasing melanosome transfer efficiency. Furthermore, Vitamin C inhibits activation of the MAPK/ERK signaling pathway by neutralizing ultraviolet-induced ROS, subsequently downregulating *MITF* gene expression and reducing TYR synthesis [72] (Figure 4). However, the oxidative-antioxidative balance within the organism is a key regulatory factor for melanin synthesis. In Taihe Silky Fowls, vitamin C scavenges free radicals and mitigates oxidative stress damage to melanocytes, aiding in the maintenance of their normal physiological function. Additionally, vitamin C promotes collagen synthesis and enhances extracellular matrix stability, providing structural support for melanocytes. Vitamin C can inhibit melanin production by blocking melanin oxidation and reducing melanin synthesis intermediates [73]; it also acts as a cofactor for hydroxylases to ensure the activity of enzymes related to melanin synthesis and stimulates the immune system to reduce abnormal plumage color in chickens caused by infections [74]. Although vitamin C suppresses melanogenesis, its role in protecting cells from oxidative damage and maintaining microenvironmental homeostasis is indispensable. Gous and Morris [75] noted that poultry under stress conditions exhibit a significantly increased demand for vitamin C which exceeds their endogenous synthesis capacity. Therefore, dietary supplementation is essential to meet requirements and sustain normal physiological function.

#### Vitamin D

**Figure 5:**
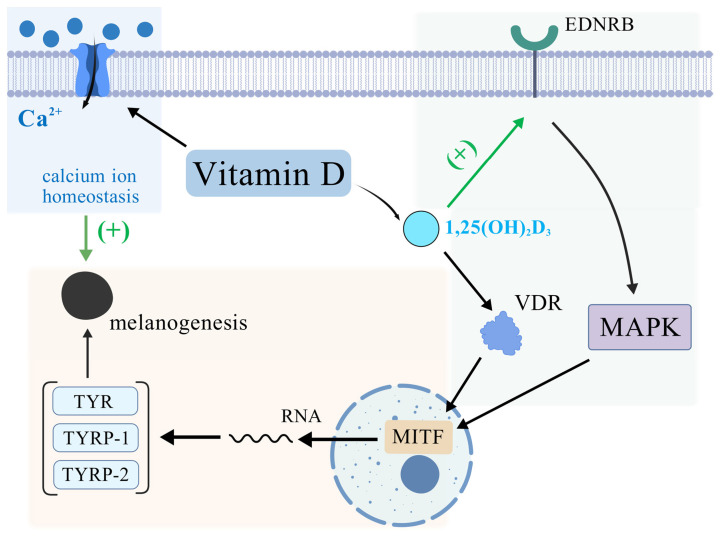
Diagram of the vitamin D regulatory mechanism. Vitamin D_3_ regulates calcium-binding proteins to improve intracellular calcium ion homeostasis and promote melanosome maturation. Simultaneously, its primary active metabolic form, 1,25-dihydroxyvitamin D3 (1,25(OH)_2_D_3_), activates the vitamin D receptor (VDR) or endothelin receptor (EDNRB) signalling pathways. By modulating MITF expression, it influences melanin formation. Data from Osborne and Hutchinson [77]. Created with BioGDP.com [111].

Vitamin D is metabolized through hepatic and renal pathways into its primary active form, 1,25-dihydroxyvitamin D_3_, which directly regulates melanogenesis by activating the vitamin D receptor (VDR) in melanocytes. As a member of the steroid hormone receptor superfamily, activated VDR binds to DNA response elements, upregulates MITF expression, enhances TYR activity, and promotes melanin synthesis. In mammals, the interaction between VDR and MITF enhances transcriptional activity through chromatin remodeling, directly regulating TYR gene expression [76]. Vitamin D_3_ also improves calcium ion homeostasis in melanocytes by modulating calcium-binding proteins, thereby facilitating melanosome maturation [77] (Figure 5). Additionally, 1,25(OH)_2_D_3_ activates the EDNRB signaling pathway, induces differentiation of melanocyte precursors, and synergistically promotes MITF nuclear translocation and downstream gene expression via MAPK/ERK phosphorylation [78]. Beyond the direct pathway of vitamin D promoting melanin synthesis, vitamin D also contributes through immune microenvironment stabilization. These mechanisms work synergistically to ensure the sustained whole-body melanin deposition and distribution in Taihe Silky Fowls.

The immunomodulatory function of vitamin D is essential for the survival and functional stability of melanocytes. Melanocytes are vulnerable to apoptosis or functional inhibition caused by local inflammatory factors. Vitamin D mitigates damage to melanocytes by inhibiting the differentiation of helper T cells 1 and 17 (Th1/Th17) and suppressing proinflammatory cytokine release. The active vitamin D-VDR complex acts upon antigen presenting cells and activated lymphocytes, inhibiting pathogenic effector T cells while inducing regulatory T cells and cytokine production, thereby modulating the body’s immune response. 1,25(OH)_2_D_3_ also influences transcriptional regulation downstream of cytokine genes. It interacts with factors such as nuclear factor of activated T cells (NFAT) and NF κB, affecting promoter complex stability and consequently regulating gene transcription. Furthermore, its effects on cytokines are dose dependent, and it can inhibit dendritic cell maturation, impacting immune responses. Li et al [79] demonstrated that supplementing basal diets with 6,400 IU/kg additional vitamin D_3_ upregulated the relative expression levels of *IL-2* and *IL-18* genes in tissues of Taihe Silky Fowls, which facilitates the regulation of the local immune microenvironment, creating favorable conditions for melanocyte activation and melanin synthesis.

#### Vitamin E

The vitamin E family, primarily comprising four isomers (α, β, γ, and δ tocopherol), constitutes essential lipid soluble antioxidants within organisms [80]. Its core antioxidant mechanism involves donating hydrogen atoms to lipid peroxyl radicals (LOO·) or alkoxyl radicals (LO·) generated during lipid peroxidation chain reactions. This action efficiently terminates the proliferative damage caused by free radicals, safeguarding critical biomolecules such as cell membrane phospholipid bilayers and nucleic acids from oxidative damage. This protective function is not isolated. Vitamin E operates within a sophisticated synergistic defense network alongside enzymatic antioxidants like superoxide dismutase (SOD) and glutathione peroxidase (GPx) [81]. The SOD catalyzes the conversion of superoxide anions to hydrogen peroxide, which GPx subsequently reduces to water using glutathione. Within this system, vitamin E primarily scavenges lipid peroxidation products, collectively forming a cascade protective barrier. At the animal physiological level, vitamin E supplementation significantly enhances T cell mediated specific immune function. The underlying mechanisms include promoting T cell differentiation and maturation within the thymus and stimulating the proliferative response of peripheral lymphocytes [82]. He [83] demonstrated that, similar to vitamin C, supplementing Vitamin E also aids in promoting homeostasis recovery in Taihe Silky Fowls under heat stress by clearing excessive ROS.

**Figure 6:**
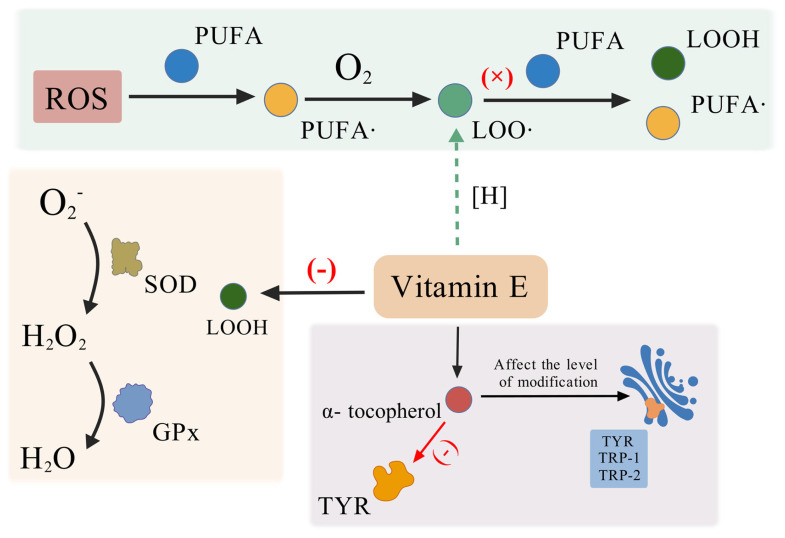
Diagram of the Vitamin E regulatory mechanism. Vitamin E can donate hydrogen atoms to lipid oxygen radicals (LOO·) or lipid peroxy radicals (LO·), thereby interrupting radical proliferation and exerting a protective effect. Simultaneously, it forms a synergistic defence network with enzymatic antioxidants such as superoxide dismutase (SOD) and glutathione peroxidase (GPx). As the primary biologically active form of Vitamin E, α-tocopherol can directly inactivate TYR or influence the post-translational modification levels of key enzyme proteins including TYR, TRP-1, and TRP-2, thereby disrupting melanin synthesis. Data from Watabe et al [78]. Created with BioGDP.com [111].

Vitamin E, leveraging its potent antioxidant properties, effectively intervenes in the complex biochemical cascade of melanin synthesis. It primarily inhibits TYR activity, reducing catalytic efficiency. Secondarily, acting as an electron donor, it promotes the reduction of dopa and DQ, thereby blocking their conversion pathway into melanin aggregates. Concurrently, vitamin E analogues, a class of structurally related antioxidant compounds, also exhibit significant capacity to inhibit melanin synthesis. Among these, α-tocopherol, the primary biologically active form of vitamin E, possesses anti-melanogenic capability. Funasaka et al [84] demonstrated that α-tocopherol not only directly inactivates TYR but may also influence the post-translational modification levels, such as glycosylation or phosphorylation, of key enzymatic proteins including TYR, TRP-1, and TRP-2, consequently interfering with their functional activity (Figure 6). Beyond α-tocopherol, other Vitamin E family members, such as γ-tocopherol, may effectively suppress melanin biosynthesis within pigment cells. This suppression occurs either through direct inhibition of TYR activity or by downregulating the mRNA translation efficiency of the TYR gene and its related enzyme genes [85].

### Minerals

#### Calcium (Ca)

Calcium participates in the biosynthesis of L-tyrosine by regulating the activity of PAH, a key rate-limiting enzyme in melanin synthesis that initiates the metabolic pathway from L-phenylalanine [86], indirectly influencing melanogenesis in Taihe Silky Fowls. Additionally, intracellular Ca^2+^ concentration, membrane Ca^2+^ channels, Ca^2+^-binding proteins, and the Wnt/Ca^2+^ signaling pathway are implicated in melanogenesis [87]. The regulatory mechanism of the Wnt/Ca^2+^ pathway involves the binding of Wnt ligands to Frizzled receptors, activating the downstream G protein-phospholipase Cβ (PLCβ) axis. PLCβ catalyzes the hydrolysis of phosphatidylinositol-4,5-bisphosphate (PIP_2_) to generate inositol triphosphate (IP_3_). IP_3_ binds to endoplasmic reticulum IP_3_ receptors, triggering Ca^2+^ release from intracellular stores and inducing transient cytosolic Ca^2+^ elevation. Increased cytosolic Ca^2+^ promotes Ca^2+^/calmodulin complex formation, which activates Ca^2+^-dependent signaling molecules such as calcineurin. Calcineurin dephosphorylates NFAT, exposing nuclear localization sequences and enabling NFAT nuclear translocation to regulate melanogenesis-related genes (e.g., *TYR*, *TYRP1*). Xu [88] demonstrated that blocking Ca^2+^ channels in melanocytes with verapamil (a Ca^2+^ antagonist) reduced intracellular Ca^2+^ levels, significantly decreasing melanin content and *TYRP1* expression. However, residual melanin levels exceeded controls, suggesting that L-type Ca^2+^ channels are the primary pathway for Ca^2+^ uptake in melanin synthesis, though alternative mechanisms may exist.

**Figure 7:**
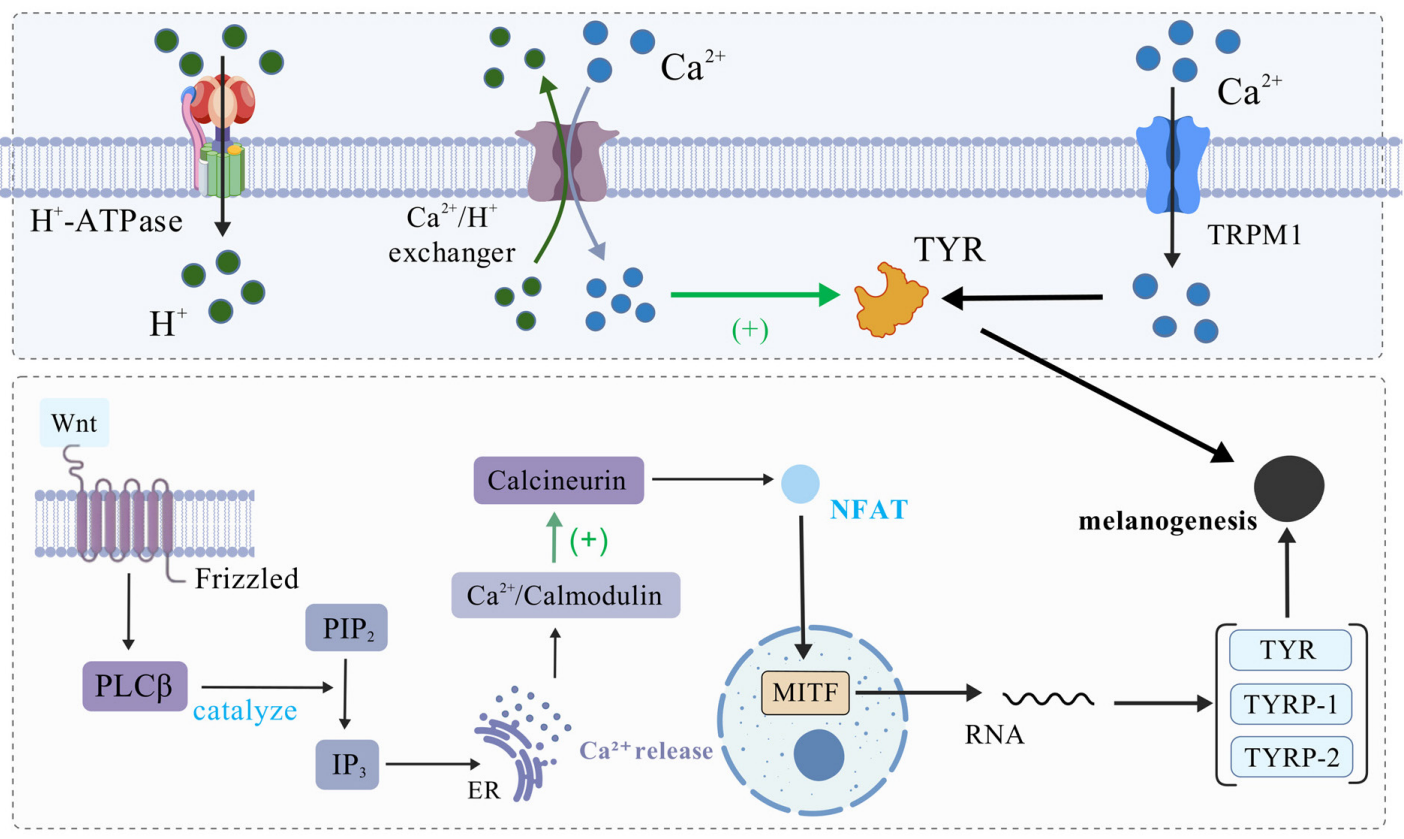
Diagram of the calcium regulatory mechanism. Calcium ions regulate intracellular calcium concentrations through interactions with relevant receptors on the cell membrane, thereby enhancing tyrosinase activity and triggering melanin transfer. Within the Wnt/Ca^2+^ pathway, Wnt ligands undergo a series of reactions to generate Ca^2+^/calmodulin complexes, exposing the nuclear localisation sequence of the nuclear factor of activated T cells (NFAT) transcription factor. This facilitates its translocation to the cell nucleus, where it modulates the expression of genes associated with melanin synthesis. Data from Xu [88]. Created with BioGDP.com [111].

In skin, Ca^2+^ critically regulates keratinocyte proliferation and differentiation. The establishment of Ca^2+^ and H^+^ concentration gradients within melanosomes is essential for melanogenesis [89]. The H^+^-ATPase pumps H^+^ to create an acidic microenvironment (pH 4.5–5.0), while Ca^2+^/H^+^ exchangers or Ca^2+^-ATPases regulate Ca^2+^ concentrations to activate TYR catalytic activity. The Ca^2+^ also induces filopodia formation in melanocyte dendrites and dose-dependently promotes melanin transfer [90]. Pigmentation may be modulated by Ca^2+^ channel activity, which increases membrane Ca^2+^ influx and alters membrane potential. Influxed Ca^2+^ is transported into melanosomes to enhance TYR activity or activates PKCβ to mediate organelle interactions [91]. The sustained Ca^2+^ influx mediated by the transient receptor potential melastatin 1 (TRPM1) channel is essential for maintaining TYR activity. Suppression of TRPM1 expression reduces both intracellular and extracellular Ca^2+^ uptake, concurrently diminishing the activity of TYR and melanin pigmentation [92]. Furthermore, melanin is closely linked to intracellular Ca^2+^ homeostasis, as it can chelate Ca^2+^ to modulate cytosolic Ca^2+^ levels [93] (Figure 7). Hoogduijn et al [94] found that melanin-Ca^2+^ complexes protect skin by mitigating H_2_O_2_-induced DNA damage in melanocytes and the stratum corneum. Xu Deli [88] demonstrated that Ca^2+^ concentrations ranging from 0.1 to 6.4 mmol/L significantly stimulated the growth of melanocytes derived from Taihe Silky Fowls, with the peak stimulatory effect observed at 1.6 mmol/L. Furthermore, 0.4 mmol/L CaCl_2_ significantly promoted melanocyte migration. Elevated Ca^2+^ concentrations (0.4 to 1.6 mM) also significantly enhanced tyrosinase activation. These effects collectively enhance melanin synthesis.

#### Selenium (Se)

Selenium is an essential trace element for animal growth, metabolism, and reproduction, primarily functioning as an antioxidant, alleviating stress, and protecting biomembrane structures *in vivo*. In nature, Se exists predominantly in organic and inorganic forms. Inorganic Se (e.g., selenates and selenites) is commonly utilized as a dietary Se source. It is passively absorbed in the intestine and reduced to selenides. Organic Se, including selenomethionine, selenocystine, and selenocysteine, is absorbed via amino acid transport mechanisms. After entering the body, Se is primarily absorbed in the duodenum. The absorbed Se initially enters the plasma and is subsequently transported to various tissues, with distribution levels varying depending on tissue type and Se intake. Generally, higher Se concentrations are observed in the kidneys, liver, pancreas, pituitary gland, and hair of animals, while lower levels are found in muscles, bones, and blood, with adipose tissue containing the least. Xiaowen et al [95] demonstrated that supplementing basal diets of 9- to 12-week-old Taihe Silky Fowls with additional selenium at 0, 0.1, 0.3, 0.5, 0.7, or 0.9 mg/kg resulted in an initial increase followed by a decrease in melanin content across tissues. Specifically, hepatic and pectoral muscle melanin peaked at 0.5 mg/kg additional selenium supplementation, while renal and dermal melanin peaked at 0.3 mg/kg.

**Figure 8:**
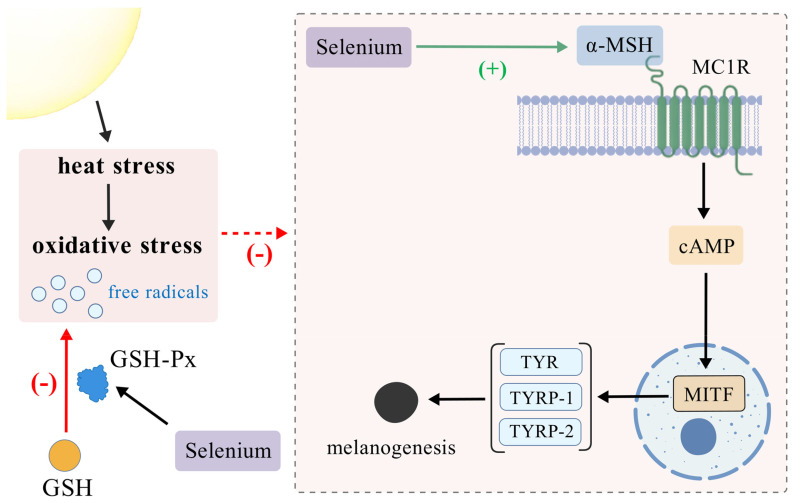
Diagram of the selenium regulatory mechanism. Under heat stress conditions, cells generate free radicals. Selenium, as an essential component of the active center of glutathione peroxidase (GSH-Px), enhances enzyme activity, promoting its catalysis of reduced glutathione (GSH) to eliminate peroxides and reduce oxidative damage. Concurrently, heat stress impairs melanin synthesis pathways (the α-MSH-MC1R-cAMP-TRP pathway), and appropriate selenium supplementation can reverse this detrimental effect. Data from Xu [97]. Created with BioGDP.com [111]. α-MSH, α-melanocyte-stimulating hormone; MC1R, melanocortin-1 receptor; cAMP, cyclic AMP.

Selenium serves as an essential component of the active site of GPx, which catalyzes the reduction of peroxides by GSH, thereby mitigating oxidative damage [96]. In Taihe Silky Fowls, Se enhances the activity of antioxidant enzymes such as GPx, effectively scavenging free radicals and reducing heat stress-induced damage to melanocytes, thereby maintaining stable melanin deposition. The mechanism by which heat stress affects melanin synthesis in Taihe Silky Fowls primarily involves the pathway: heat stress → oxidative stress → expression of melanin-related genes (α-MSH – MC1R – cAMP – TRP signaling cascade) → melanin synthesis. Specifically, heat stress tends to reduce the secretion and expression of α-MSH, MC1R, and cAMP within the melanin synthesis pathway in Taihe Silky Fowls. Conversely, dietary supplementation with appropriate levels of additional selenium can reverse the adverse effects of chronic heat stress on molecules related to melanin synthesis in these fowls. This reversal is achieved by promoting the secretion of α-MSH and the expression of enzymes such as TYRP (Figure 8). Xu [97] demonstrated that dietary additional selenium supplementation at an optimal level of 0.5 mg/kg to 0.7 mg/kg mitigated the effects of heat stress on the production performance and melanin synthesis in Taihe Silky Fowls. Li et al [98] reported that supplementing basal diets of Taihe Silky Fowls with selenium at levels of 0, 0.10, 0.30, 0.50, 0.70, and 0.90 mg/kg resulted in significantly enhanced GPx activity, TYR activity, and melanin deposition in silky fowl tissues when selenium was added within the range of 0.10 mg/kg to 0.70 mg/kg.

#### Copper (Cu)

**Figure 9:**
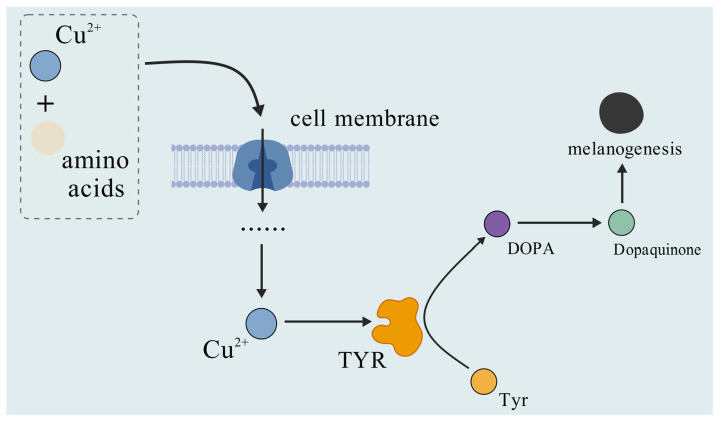
Diagram of the copper regulatory mechanism. Copper combines with amino acids to form chelates, which enter the cell and after a series of reactions become cofactors for tyrosinase, enabling it to catalyze the conversion of tyrosine to dopa, which after a series of reactions forms dopaquinone and synthesizes melanin. Data from Guo et al [101]. Created with BioGDP.com [111].

Copper is widely utilized as a dietary additive in the poultry industry, serving functions such as growth promotion, anti-inflammatory and antibacterial activity, antioxidant effects, and participation in skeletal development. Notably, it plays a critical role in melanin formation [99]. Tyrosinase, also known as polyphenol oxidase, is a metalloenzyme utilizing Cu^2+^ as its prosthetic group. Its active center comprises two copper ion binding sites, and the prosthetic copper ion is essential for enzyme activity [100]. Dietary copper deficiency reduces tyrosinase activity in animals. This subsequently impedes the catalytic conversion of tyrosine to melanin, leading to decreased melanin synthesis [101] (Figure 9). Chen et al [102] found that copper ions at certain concentrations significantly enhance TYR activity. However, beyond a specific concentration range, copper ions inhibit TYR activity, with the inhibitory effect intensifying as copper ion concentration increases. Xu et al [103] demonstrated that supplementing the diet of 5- to 8-week-old Taihe Silky Fowls with additional copper at levels of 0, 5, 15, 30, 60, and 125 mg/kg resulted in tissue melanin content initially increasing and then decreasing as copper supplementation increased. Both serum and tissue TYR activity, along with tissue melanin content, peaked at the 30 mg/kg supplementation level.

Organically synthesized copper sources exhibit superior advantages over inorganic copper sources in terms of bioavailability, enhancement of meat and egg quality, reduction of toxic side effects, and mitigation of environmental pollution [104]. For example, amino acid-chelated copper, classified as a third-generation trace element feed additive, includes chelated and complexed copper salts. Amino acid-chelated copper possesses stable chemical properties. Upon chelation with amino acids, the internal charge of the molecule becomes neutralized. The five-membered and six-membered rings formed by copper-amino acid chelates exhibit high stability, preventing adsorption onto insoluble colloids that hinder elemental absorption and minimizing interference from other inorganic salts or antagonistic substances. In conventional diets, the absorption rate of trace elements in chelated forms is 1.8 to 4.0 times higher than that of inorganic forms [105]. Wu et al [106] revealed that supplementing diets with appropriate levels of additional amino acid-chelated copper enhances growth performance, tissue melanin content, antioxidant capacity, and reduces cellular damage in Guangxi silky fowls.

#### Iron (Fe)

Within cells, iron primarily exists in two valence states: Fe^2+^ (reduced) and Fe^3+^ (oxidized). It participates in physiological processes including signal transduction, oxygen transport, and energy metabolism. The metabolic fates of intracellular iron include: (1) Entry into mitochondria to maintain normal physiological metabolism; (2) Storage within ferritin; (3) Efflux to the extracellular space via the membrane transporter ferroportin [107]. Iron absorbed from the diet via the duodenum enters the bloodstream and is preferentially stored in the liver. It is subsequently transported to tissues by transferrin (Tf). The Fe^3+^ binds to Tf, forming Tf-bound iron (TBI). This complex binds to Tf receptor 1 (TfR1) on the cell membrane and enters the cell via endocytosis. The acidic environment within the endocytic vesicle facilitates the reduction of Fe^3+^ to Fe^2+^ by reductases. The Fe^2+^ is then released into the cytosol by divalent metal transporter 1 (DMT1) [108]. However, iron metabolism imbalance triggers a critical shift: Excess Fe^2+^ generates highly reactive •OH via the Fenton reaction, initiating lethal peroxidation of unsaturated fatty acids in membrane lipids. When accumulated lipid peroxides exceed the repair capacity threshold of GPx 4 (GPx4), ferroptosis is induced [109]. This process involves disintegration of cell membrane integrity and complete collapse of the melanin synthesis machinery, ultimately leading to potentially irreversible disruption of melanin deposition.

**Figure 10:**
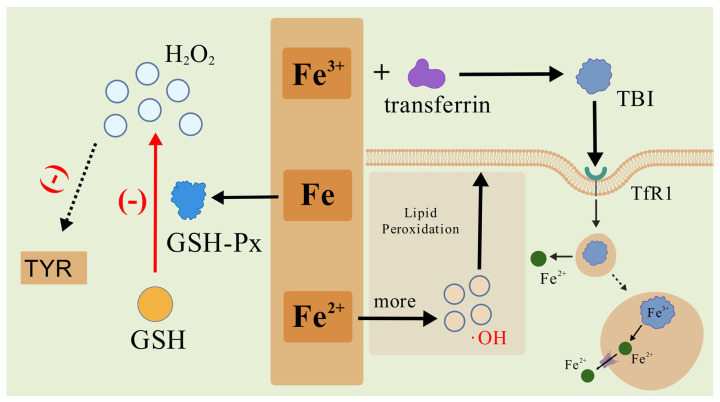
Diagram of the iron regulatory mechanism. Fe^3+^ binds with transferrin to form transferrin-bound iron (TBI). Upon entering cells, it is reduced to Fe^2+^ within the acidic environment of endosomal vesicles and released into the cytoplasm. Concurrently, iron serves as a stabilizing factor for the active center of glutathione peroxidase (GSH-Px), ensuring its efficient catalysis of the reduction of harmful lipid peroxides (such as H_2_O_2_) by reduced glutathione (GSH). Data from Xu [88]. Created with BioGDP.com [111].

Iron regulates melanin deposition in Taihe Silky Fowls primarily through its precise control of redox homeostasis. Within physiological concentration ranges, Fe^2+^/Fe^3+^ significantly reduces ROS levels within melanocytes by activating the core antioxidant defense system centered on GPx. The mechanism involves iron stabilizing the selenocysteine residue within the GPx active site. This stabilization ensures the enzyme efficiently catalyzes the reduction of harmful lipid peroxides, such as H_2_O_2_, into harmless water and alcohols using reduced GSH. The resulting ROS clearance directly mitigates oxidative inactivation damage to tyrosinase, thereby sustaining its catalytic activity (Figure 10). Xu [88] demonstrated that in cultured Taihe Silky Fowl melanocytes, specific concentrations of both Fe^2+^ and Fe^3+^ individually decrease intracellular ROS content, promote TYR activation, and stimulate melanocyte growth. Furthermore, the combined application of Fe^2++^ and Fe^3+^ promotes TYRP1 expression in these melanocytes.

## CONCLUSION

This paper reviews the underlying mechanisms of the black pigmentation trait in Taihe Silky Fowls. It focuses primarily on how specific nutrients (amino acids, vitamins, and minerals) regulate melanin biosynthesis and deposition at the cellular pathway level, while also discussing their optimal dietary inclusion levels for practical production. This paper contributes to a deeper understanding of melanin deposition patterns in these fowls and lays theoretical groundwork for scientifically formulating their diets to enhance their significant nutritional and medicinal properties. Future research needs to elucidate the molecular details of nutrient regulation, particularly as the specific influencing mechanisms require further investigation, to facilitate the efficient development and utilization of the distinctive traits of Taihe Silky Fowls.

## Data Availability

Upon reasonable request, the datasets of this study can be available from the corresponding author.
